# First diagnosis of thrombotic thrombocytopenic purpura after SARS-CoV-2 vaccine – case report

**DOI:** 10.1186/s12882-021-02616-3

**Published:** 2021-12-11

**Authors:** Bilgin Osmanodja, Adrian Schreiber, Eva Schrezenmeier, Evelyn Seelow

**Affiliations:** 1 Department of Nephrology and Medical Intensive Care, Charité – Universitätsmedizin Berlin, corporate member of Freie Universität Berlin, Humboldt-Universität zu Berlin, and Berlin Institute of Health, Charitéplatz 1, 10117 Berlin, Germany; 2 Berlin Institute of Health (BIH), Anna-Louisa-Karsch-Str. 2, 10178 Berlin, Germany

**Keywords:** Purpura, Thrombotic thrombocytopenic, Thrombocytopenia, COVID-19 vaccines, Plasma exchange, Case report

## Abstract

**Background:**

We report a case of a 25-year-old male patient, who developed acquired thrombotic thrombocytopenic purpura (aTTP) after receiving a first dose of mRNA-based SARS-CoV-2 vaccine Spikevax (mRNA-1273, Moderna Biotech, USA). While this is the first case in literature describing a case of aTTP after receiving the Spikevax vaccine, there are two other cases after mRNA-based Covid-19 vaccine and two after adenoviral SARS-CoV-2 vaccine.

**Case presentation:**

The patient presented with persisting malaise, fever, headache, word-finding difficulties, nausea, vomiting, petechial bleeding, and hematuria 13 days after receiving a first dose of vaccination. Laboratory testing showed low platelet count, Coombs-negative hemolytic anemia, and mild acute kidney injury. We excluded vaccine induced immune thrombotic thrombocytopenia (VITT) as another important differential diagnosis and the final diagnosis was established after ADAMTS-13 (A Disintegrin And Metalloproteinase with a ThromboSpondin type 1 motif, member 13) activity was found to be < 1% (reference range > 40%) and ADAMTS-13 antibodies being 72.2 IU/L (reference range < 12 IU/L).

We initiated empiric therapy of plasmapheresis and corticosteroids on admission and started caplacizumab the day after. The patient’s thrombocyte count normalized 3 days after admission, hemolysis and acute kidney injury resolved after 2 weeks. The patient received 2 doses of rituximab (1 g each) after the diagnosis of immune TTP was established. One month after the initial presentation, the patient is in good overall condition, but still receives daily caplacizumab due to ADAMTS-13 activity of < 1%.

**Conclusions:**

Low platelet count after vaccination against SARS-CoV-2 has gained attraction after vaccine-induced immune thrombotic thrombocytopenia (VITT) has been described as a rare but severe complication of adenoviral-based vaccines. Thrombotic thrombocytopenic purpura (TTP) is an important differential diagnosis, but there are only few reports of TTP following SARS-CoV-2 vaccination. Despite pathophysiological and clinical differences of both entities, diagnostic uncertainty can result in the acute setting, since they share main symptoms such as headache and neurological alterations in addition to thrombocytopenia. In difference to other cases reported, this patient developed first symptoms of TTP as early as 4 days after vaccination, which suggests that vaccination merely acted as trigger for occult TTP, instead of truly inducing an autoimmunological process.

## Background

Thrombotic thrombocytopenic purpura (TTP) is a rare thrombotic microangiopathy caused by decreased activity of ADAMTS-13 (A Disintegrin And Metalloproteinase with a ThromboSpondin type 1 motif, member 13), which leads to disseminated thrombus formation [1]. Most frequently, autoantibody formation against ADAMTS-13 leads to acquired TTP (aTTP), but in rare cases, mutations of the ADAMTS-13 gene can lead to congenital TTP as well [1, 2]. While natural disease course shows high mortality of > 90% due to ischemic kidney, brain and heart injury, therapy with plasma exchange, caplacizumab and immunosuppression decreased mortality to < 20% [3]. A variety of causative events, as well as triggers for a disease episode have been described in the literature, including vaccination against viral diseases.

We report the case of a 25-year-old male patient with unremarkable previous medical history, who developed a first episode of acquired thrombotic thrombocytopenic purpura (aTTP) after receiving a first dose of SARS-CoV-2 vaccine Spikevax (mRNA-1273, Moderna Biotech, USA) [4]. While this is the first case in literature describing a case of aTTP after receiving the Spikevax vaccine, we found two other cases after mRNA-based SARS-CoV-2 vaccine (Pfizer-BioNTech) and two after adenoviral SARS-CoV-2 vaccine (AstraZeneca). Since aTTP and the more common adverse event of adenoviral SARS-CoV-2 vaccines – vaccine induced immune thrombocytopenia (VITT) share common clinical and laboratory findings, distinguishing both is a challenge in the acute setting [5].

This case report follows CARE (Case Report) Guidelines, and a checklist is available with this article.

## Case presentation

A 25-year-old male patient (175 cm, 107 kg) was admitted to our university hospital, after he presented in another emergency department 13 days after receiving a first dose of mRNA-based SARS-CoV-2 vaccination Spikevax (mRNA-1273, Moderna Biotech, USA). Chronologically, the patient’s symptoms developed as follows: immediately after vaccination he developed pain at the injection site. Additionally, he developed headache 2 days after the vaccination, and fever of up to 38.7 °C after 3 days. To ameliorate the fever, he took 500 mg of acetaminophen once. On day 4, he developed what he called a “rash” covering his abdomen and both legs. The same day, his urine color changed towards an “orange” tone and became foul smelling. During the following days, headache and fever waned, but progressive fatigue as well as exertional dyspnea developed. On the day of admission, he still had malaise, and woke up with a severe headache (numeric rating scale 7/10). That day, he vomited once with an empty stomach, and had persisting nausea the remaining day. Later in the other emergency department, he experienced word-finding difficulties, which he attributed to anxiety. He denied taking any other medication except for acetaminophen, drinking alcohol or using illicit drugs, which could have triggered the episode of aTTP. He had no family history for hematological, autoimmunological or kidney diseases.

On the initial examination, the only notable finding was petechiae on both legs, which was the “rash” the patient described. The initial laboratory diagnostic showed Coombs-negative hemolytic anemia (Hb 7.4 g/dL, LDH 999 U/L, schistocyte count 2.1%, haptoglobin < 0.1 g/L), low platelet count (29/nL), and elevated creatinine (1.5 mg/dL, eGFR 63.8 mL/min/1.73m^2^).

In summary, thrombotic microangiopathy was present, and a presumptive diagnosis of aTTP was made. This was supported by high PLASMIC score of 6 points, predicting a high risk of 72% of ADAMTS-13 (A Disintegrin And Metalloproteinase with a ThromboSpondin type 1 motif, member 13) deficiency. PLASMIC score was developed to identify those patients with low platelet count and signs of microangiopathic hemolytic anemia (MAHA), who have low ADAMTS-13 activity < 10%. This is important, because those patient will benefit from immediate plasma exchange treatment [6].

Due to the combination of SARS-CoV-2 vaccine, headache and thrombocytopenia, an important differential diagnosis was vaccine-induced immune thrombotic thrombocytopenia (VITT), which is described as a rare adverse event of adenoviral vector vaccines, but not mRNA-based vaccines against SARS-CoV-2. Since neurological examination and head computer tomography were unremarkable, and no clinical signs of cerebral or peripheral thrombosis were present, we did not anticoagulate the patient with argatroban, but only performed routine testing for heparin-induced thrombocytopenia (Milenia QuickLine HIT-Test, Milenia Biotech, Gießen, Germany), and sent out additional specimens for heparine-induced platelet activation (HIPA) and platelet-factor 4 enhanced platelet-activation test (PIPA), which are described elsewhere [5, 7]. All tests returned negative. Additionally, cranial magnetic resonance imaging including magnetic resonance angiography were performed to exclude cerebral sinus venous thrombosis (CSVT). Secondary reasons for TTP such as viral infections, autoimmune disorders, hematological or solid organ malignancies, as well as Shigatoxin-producing enterohemorrhagic *E. coli* as trigger for typical hemolytic uremic syndrome (HUS) were ruled out. Complement investigations showed normal C3 and C4 levels and ruled out anti-factor H antibodies. Functional assays showed normal activity of the classical and the alternative pathway. A mild increase of soluble terminal complement activation fragments (C5b-9) levels indicated concomitant low grade complement activation. In conclusion, we classified this as autoimmune TTP.

**Fig. 1:**
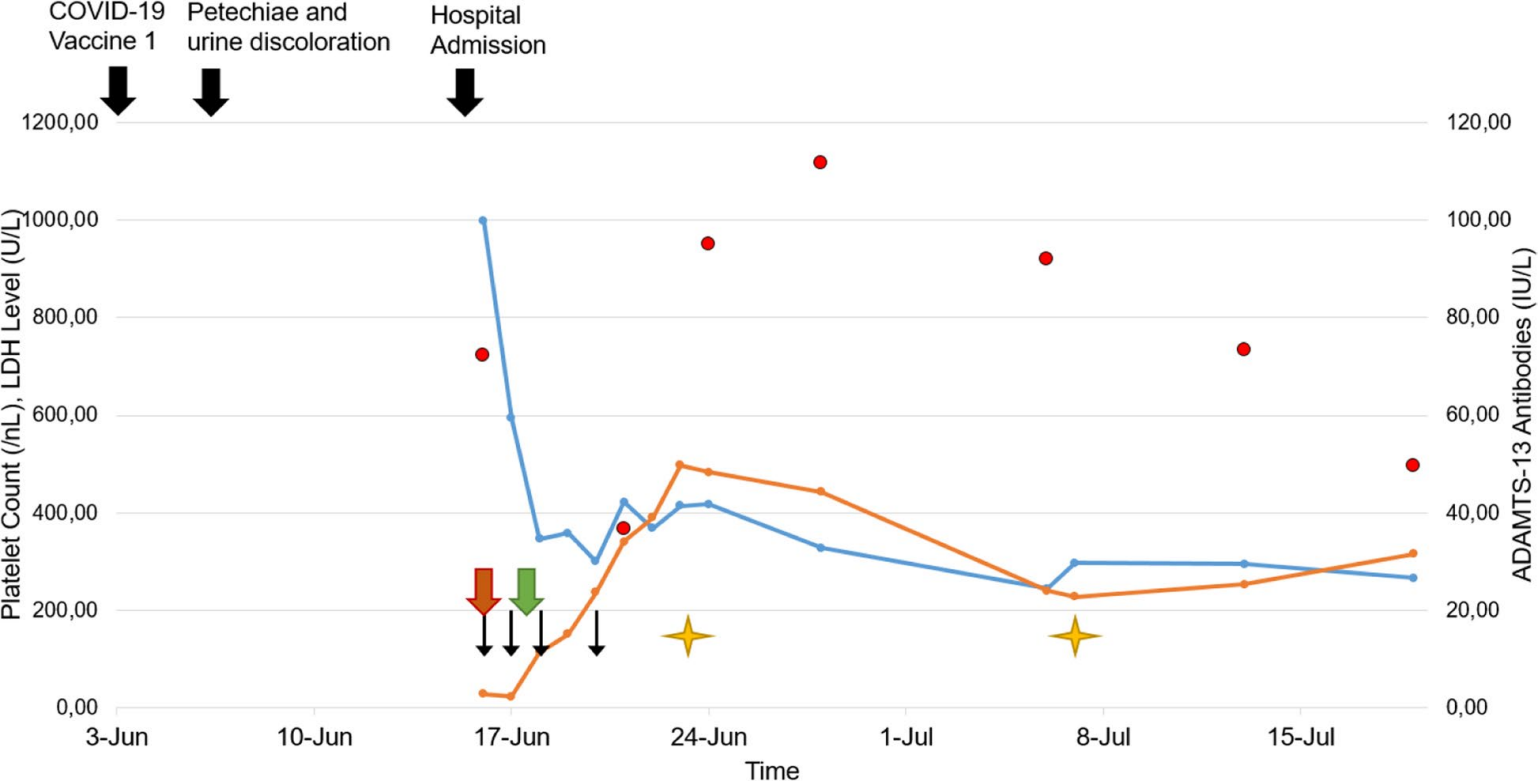
Main events in chronological order. Thick black arrow – clinical event, thin black arrow – plasma exchange, red arrow – start steroids, green arrow – start caplacizumab, star – rituximab, blue line – LDH Level (U/L; NV 135-250), orange line – platelet count (/nL; NV 150-370), red dot – ADAMTS-13 antibodies (IU/mL; NV<12), LDH – Lactate Dehydrogenase; NV – Normal Values; Jun – June; Jul – July

After a suspected diagnosis of aTTP was made, the patient received daily plasma exchange with substitution of 2.5 L fresh frozen plasma and steroid pulse therapy of 250 mg prednisolone daily for the first 3 days after admission. Additionally, caplacizumab (10 mg s.c. once daily) was started the second day after admission and continued until now (day 46). Plasma exchange was discontinued after 4 treatments since thrombocyte count normalized on day 3. Steroid therapy was tapered quickly to currently 6 mg daily. One week after admission, ADAMTS-13 activity returned highly suppressed (< 1%), with highly elevated ADAMTS-13 antibodies (72.2 IU/mL) as well as decreased ADAMTS-13 antigen, which confirmed the suspected diagnosis. Since ADAMTS-13 antibodies were detected, a diagnosis of autoimmune TTP was made, and immunosuppressive therapy with rituximab (1000 mg) was initiated 1 week after admission and repeated 2 weeks later. The patient was discharged 8 days after initial admission and is in continuous follow-up in our outpatient clinic. Hemolysis stopped after 12 days, and creatinine stabilized at 1 mg/dl after 2 weeks of therapy. The main clinical events, diagnostic and therapeutic measures are summarized in Fig.1. Additional medical therapy includes candesartan (4 mg b.i.d.) for arterial hypertension, Vitamin D (1000 IE once daily) and pantoprazole (20 mg once daily) for prophylaxis against osteoporosis and gastric ulcer as well as cotrimoxazole for prophylaxis against pneumocystis pneumonia. Over a month after initial admission, the patient is in good general health and most laboratory values normalized as shown in Table 1. Clinical signs of TTP such as petechiae dissolved and did not reappear. Since ADAMTS-13 activity is still < 1%, the patient is still receiving caplacizumab once daily [8]. Since ADAMTS-13 antigen is severely diminished, we suspect that the patient developed depleting ADAMTS-13 antibodies [1].

**Table 1:** Important laboratory tests in chronological order

Laboratory Test	Day 0	Day 1	Day 2	Day 3	Day 5	Day 7	Day 12	Day 20	Day 27	Day 33
Hemoglobin level (g/dL; NV 13.5–17)	7.40	7.20	6.60	7.30	8.10	9.10	11.1	13.1	14.6	14.0
Platelet count (/nL; NV 150–370)	29.00	23.00	114	151	341	498	443	240	253	316
Leukocyte count (/nL; NV 3.9–10.5)	15.08	11.60	16.42	11.15	21.37	25.57	9.75	10.42	10.35	10.0
Lymphocyte count (/nL; NV 1.1–4.5)	2.40	1.47	3.82	1.62	3.65	1.45		0.95		
Reticulocyte count (/nL; NV 25–105)	233.1	233.8		262.4	277.2	424.9				
Schistocyte count (%; NV < 1.0)	2.1%									
LDH level (U/L; NV 135–250)	999	594	347	359	422	415	328	245	295	267
Haptoglobin level (g/L; NV 0.3–2.0)	< 0.10	0.31	0.30	0.30	0.20	< 0.10	0.70	1.00	1.31	1.25
Bilirubin indirect (g/dL; < 0.75)	1.91	1.00	0.30	0.29	0.22	0.24		0.21		
D-Dimers (mg/L; NV < 0.5)	2.95				4.30					
CRP (mg/L; NV < 5.0)	9.50	7.00	6.20	6.90	2.30	5.60	0.90	0.50	2.90	0.70
Creatinine (mg/dL; NV 0.70–1.20)	1.50	1.28		1.33	1.24	1.14	1.01	0.96	0.98	0.86
ADAMTS-13 Antibodies (IU/mL; NV < 12)	72.2				36.7		111.6	> 92.0	73.2	49.7
ADAMTS-13 Antigen (%; NV > 40)	5				< 1		< 1	< 1	3	6

The patient reported full adherence to therapy, which is supported by normal thrombocyte count despite ADAMTS-13 activity < 1%. As a result of steroid treatment, the patient developed steroid acne and as a suspected adverse event of cotrimoxazole treatment developed elevated liver enzymes. Since steroid therapy is tapered rapidly no specific therapy for steroid acne was started, but cotrimoxazole was paused after 2 weeks.

We recommended to withhold second vaccination against SARS-CoV-2 for two reasons. On the one hand, the patient has a low risk for severe course due to first successful vaccination and young age. On the other hand, due to current rituximab treatment, the response to vaccination will be severely reduced at the moment.

### Patient perspective

Regarding the initial presentation, the patient described that he can neither fully remember the situation in the emergency department nor the fact that he wrote cryptic text messages to his girlfriend or had word finding difficulties. Regarding the therapeutic measures, the patient reported that his “vitality” returned after the third plasma exchange treatment, but that those treatments were uncomfortable, since he developed chills. After discharge, the patient recognized increased strength and insomnia, as well as acne, but no weight gain.

Initially, the patient was worried that he could develop further deterioration of kidney function including dialysis. Now, he is confident that he will regain his normal health with medication and is not afraid between the appointments anymore.

## Discussion and conclusions

To our knowledge, this is the first report of TTP after Spikevax vaccine, which is an important differential diagnosis in a patient with low platelet count after SARS-CoV-2 vaccine. Still, there is some doubt, whether the autoimmunological process leading to aTTP itself was induced by vaccination. As the patient developed his first symptoms of aTTP as early as 4 days after vaccination, it is unlikely that formation of autoreactive B-cells, plasma cells and autoantibodies occurred that quickly. Usually, such process takes about 7–10 days and additional time for the clinical phenotype to develop is required as well. Instead, we argue that this patient most probably had occult undiagnosed aTTP and that the acute episode was then triggered by vaccination. This must be considered for other cases of aTTP after SARS-CoV-2 vaccination as well.

We treated our patient with steroids, plasma exchange, caplacizumab and rituximab according to the ISTH (International Society on Thrombosis and Haemostasis) Guidelines for Treatment of Thrombotic Thrombocytopenic Purpura from 2020 [9]. His response to treatment did not differ from other patients with aTTP.

In the literature, two case reports after adenoviral vector vaccines and two after mRNA-based vaccine were found, searching PubMed on July 27, 2021. For one case after mRNA-based vaccine, the first symptoms of bruising developed 2 weeks after the first vaccination, but diagnostic tests were performed 2 weeks after the second vaccination, when bruising increased [10]. The second case report described a patient with underlying human immunodeficiency virus (HIV) infection, who developed symptoms leading to TTP diagnosis 1 week after receiving the second dose [11]. For the two TTP cases after adenoviral vector vaccine, symptoms started 10 days and 37 after first vaccination, respectively [12, 13]. In all cases, only timing and absence of causes for secondary TTP led to the conclusion that TTP was induced by SARS-Cov-2 vaccination. There is no causal explanation for TTP induction to this point regarding molecular similarities between the two antigens ADAMTS-13 and SARS-CoV-2-spike (S) protein. Hence, it is conceivable that in some of the reported cases, vaccination was only triggering occult undiagnosed TTP, as we suspect it in our case.

In addition to such uncertainties, the acute presentation of patients with low platelet count after SARS-CoV-2 vaccination poses a diagnostic challenge to the treating physicians. Even if VITT is an adverse event of adenoviral vector vaccines, and TTP presents with thrombotic microangiopathy instead of CSVT, diagnosis can be challenging since both entities present with low platelet count and can present with neurological symptoms such as headache or focal deficits. Diagnostic tests such as ADAMTS-13 activity for TTP or HIPA and PIPA for VITT have long turnaround times, therefore it is important to make a sound suspected diagnosis and ensure effective empiric therapy. Increased schistocyte count, Coombs-negative hemolytic anemia, acute non-severe kidney injury and PLASMIC score can point towards TTP, while CSVT on MRI as well as a history of adenoviral vector vaccine points towards VITT. With the number of case reports of TTP after SARS-CoV-2 vaccine increasing, progress needs to be made with respect to the causal relationship.

## Data Availability

The datasets used and/or analyzed during the current study are available from the corresponding author on reasonable request.

## Abbreviations

TTP: Thrombotic thrombocytopenic purpura
aTTP: Acquired thrombotic thrombocytopenic purpura
VITT: Vaccine induced immune thrombotic thrombocytopenia
ADAMTS-13: A Disintegrin And Metalloproteinase with a ThromboSpondin type 1 motif, member 13
HIPA: Heparine-induced platelet activation
PIPA: Platelet-factor 4 enhanced platelet-activation test
CSVT: Cerebral sinus venous thrombosis
HUS: Hemolytic uremic syndrome
HIV: Human immunodeficiency virus
MRI: Magnet resonance imaging

## References

[1] Kremer Hovinga JA, Coppo P, Lämmle B, Moake JL, Miyata T, Vanhoorelbeke K (2017). Thrombotic thrombocytopenic purpura. Nat Rev Dis Prim.

[2] Alwan F, Vendramin C, Liesner R (2019). Characterization and treatment of congenital thrombotic thrombocytopenic purpura. Blood.

[3] Miesbach W, Menne J, Bommer M (2019). Incidence of acquired thrombotic thrombocytopenic purpura in Germany: a hospital level study. Orphanet J Rare Dis.

[4] Baden LR, El Sahly HM, Essink B (2021). Efficacy and safety of the mRNA-1273 SARS-CoV-2 vaccine. N Engl J Med.

[5] Greinacher A, Thiele T, Warkentin TE, Weisser K, Kyrle PA, Eichinger S (2021). Thrombotic thrombocytopenia after ChAdOx1 nCov-19 vaccination. N Engl J Med.

[6] Bendapudi PK, Upadhyay V, Sun L, Marques MB, Makar RS (2017). Clinical scoring Systems in Thrombotic Microangiopathies. Semin Thromb Hemost.

[7] Greinacher A, Michels I, Kiefel V, Mueller-Eckhardt C (1991). A rapid and sensitive test for diagnosing heparin-associated thrombocytopenia. Thromb Haemost.

[8] Volker LA, Kaufeld J, Miesbach W (2020). ADAMTS13 and VWF activities guide individualized caplacizumab treatment in patients with aTTP. Blood Adv.

[9] Zheng XL, Vesely SK, Cataland SR (2020). ISTH guidelines for treatment of thrombotic thrombocytopenic purpura. J Thromb Haemost.

[10] de Bruijn S, Maes MB, De Waele L, Vanhoorelbeke K, Gadisseur A. First report of a de novo iTTP episode associated with an mRNA-based anti-COVID-19 vaccination. J Thromb Haemost. 2021. 10.1111/jth.15418. 10.1111/jth.15418 PMC8236927 34105244

[11] Waqar SHB, Khan AA, Memon S. Thrombotic thrombocytopenic purpura: a new menace after COVID bnt162b2 vaccine. Int J Hematol. 2021. 10.1007/s12185-021-03190-y. 10.1007/s12185-021-03190-y PMC8280631 34264514

[12] Al-Ahmad M, Al-Rasheed M, Shalaby NAB. Acquired thrombotic thrombocytopenic purpura with possible association with AstraZeneca-Oxford COVID-19 vaccine. EJHaem. 2021. 10.1002/jha2.219. 10.1002/jha2.219 PMC8242544 34226899

[13] Yocum A, Simon EL. Thrombotic thrombocytopenic Purpura after Ad26.COV2-S vaccination. Am J Emerg Med. 2021. 10.1016/j.ajem.2021.05.001. 10.1016/j.ajem.2021.05.001 PMC8095021 33980419

